# Synthesis and crystal structure of NaCuIn(PO_4_)_2_

**DOI:** 10.1107/S2056989020001929

**Published:** 2020-02-14

**Authors:** Elhassan Benhsina, Jamal Khmiyas, Said Ouaatta, Abderrazzak Assani, Mohamed Saadi, Lahcen El Ammari

**Affiliations:** a Laboratoire de Chimie Appliquée des Matériaux, Centre des Sciences des Matériaux, Faculty of Sciences, Mohammed V University in Rabat, Avenue Ibn Batouta, BP 1014, Rabat, Morocco; b Laboratoire de Chimie Appliquée des Matériaux, Centre des Sciences des Matériaux, Faculty of Sciences, Mohammed V University in Rabat, Avenue Ibn Batouta, B.P. 1014, Rabat, Morocco

**Keywords:** crystal structure, phosphate, *AMM*’(PO_4_)_2_ family, trigonal–bipyramidal coordination, isotypism

## Abstract

Single crystals of sodium copper(II) indium bis­[phosphate(V)], NaCuIn(PO_4_)_2_, were grown from the melt under atmospheric conditions. The title phosphate crystallizes in the space group *P*2_1_/*n* and is isotypic with KCuFe(PO_4_)_2_. In the crystal, two [CuO_5_] trigonal bipyramids share an edge to form a dimer [Cu_2_O_8_] that is connected to two PO_4_ tetra­hedra. The obtained [Cu_2_P_2_O_12_] units are inter­connected through vertices to form sheets that are sandwiched between undulating layers resulting from the junction of PO_4_ tetra­hedra and [InO_6_] octa­hedra. The two types of layers are alternately stacked along [101] and are joined into a three-dimensional framework through vertex- and edge-sharing, leaving channels parallel to the stacking direction. The channels host the sodium cations that are surrounded by four oxygen atoms in form of a distorted disphenoid.

## Abstract 2

In the crystal, edge-sharing [CuO_5_] trigonal bipyramids form dimers that are linked to PO_4_ tetra­hedra *via* a common edge. The obtained [Cu_2_P_2_O_12_] units are inter­connected through vertices to form sheets that are sandwiched between undulating layers resulting from the junction of PO_4_ tetra­hedra and [InO_6_] octa­hedra. The two types of layers are alternately stacked along [101] and are joined into a three-dimensional framework through vertex- and edge-sharing, leaving channels parallel to the stacking direction in which the Na^+^ ions are located.

## Chemical context   

Transition-metal phosphates have been the subject of intensive research as a result of their inter­esting physical properties and potential applications in wide-ranging fields such as catalysis, electrochemistry, luminescence (Tie *et al.*, 1995 ▸; Pan *et al.*, 2006 ▸; Yang *et al.*, 2016 ▸) and ion exchange (Cheetham *et al.*, 1999 ▸; Han *et al.*, 2015 ▸; Manos *et al.*, 2005 ▸, 2007 ▸; Plabst *et al.*, 2009 ▸; Stadie *et al.*, 2017 ▸). In these materials, the anionic framework is built up from PO_4_ tetra­hedra linked to different kinds of transition metal (*TM*) coordination polyhedra of the form [*TM*O_*n*_] (*n* = 4, 5 and 6), leading to a large variety of crystal structure families. This structural diversity is mainly associated with the ability of *TM* cations to adopt different oxidation states in various coordination polyhedra. Based on previous hydro­thermal investigations aimed at orthophosphates of general formula (*M*,*M*′′)_3_(PO_4_)_2_·*n*H_2_O (*M* and *M*′′ = bivalent cations), we have reported on synthesis and characterization of the phosphates Ni_2_Sr(PO_4_)_2_·2H_2_O (Assani *et al.*, 2010 ▸), Mg_1.65_Cu_1.35_(PO_4_)_2_·H_2_O (Khmiyas *et al.*, 2015 ▸) and Mn_2_Zn(PO_4_)_2_·H_2_O (Alhakmi *et al.*, 2015 ▸). In this context, the aim of the present study was to develop new phases belonging to the series *AM*′′*M*′′′(PO_4_)_2_ where *A*, *M*′′ and *M*′′′ are mono-, bi- and trivalent cations, respectively. As a result, we report here on synthesis and crystal structure of the new compound NaCuIn(PO_4_)_2_.

## Structural commentary   

**Figure 1:**
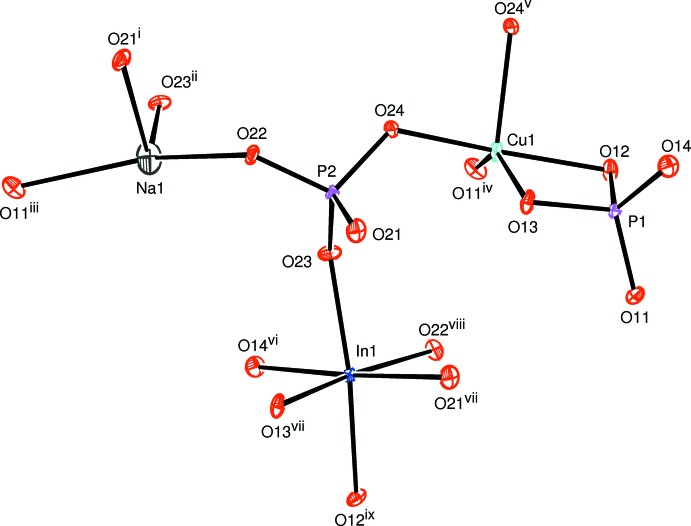
The principal building units in the crystal structure of NaCuIn(PO_4_)_2_. Displacement ellipsoids are drawn at the 50% probability level. [Symmetry codes: (i) *x* + 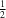, −*y* − 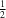, *z* + 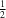; (ii) −*x* + 1, −*y*, −*z* + 2; (iii) −*x* + 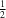, *y* − 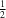, −*z* + 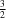; (iv) *x* + 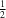, −*y* + 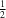, *z* + 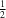; (v) −*x* + 1, −*y*, −*z* + 1; (vi) *x*, *y*, *z* + 1; (vii) −*x*, −*y*, −*z* + 1; (viii) −*x* + 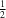, *y* + 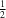, −*z* + 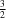; (ix) *x* − 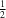, −*y* + 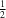, *z* + 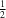.]

The principal building units of the crystal structure of NaCuIn(PO_4_)_2_ are two PO_4_ tetra­hedra linked to a [CuO_5_] triangular bipyramid [Cu—O bond-length range of 1.9088 (9) to 2.1939 (9) Å] and to an [InO_6_] octa­hedron [In—O bond lengths range from 2.1028 (10) to 2.2051 (9) Å], and is completed by a distorted [NaO_4_] polyhedron (Fig. 1 ▸). The P—O bond lengths in the two phosphate tetra­hedra are similar and comparable with those of similar phosphates. However, the P1—O distances, varying between 1.5035 (10) and 1.5729 (9) Å, indicate a somewhat higher distortion of this tetra­hedron than the P2—O distances [between 1.5297 (9) and 1.5488 (9) Å] of the other tetra­hedron.

**Figure 2:**
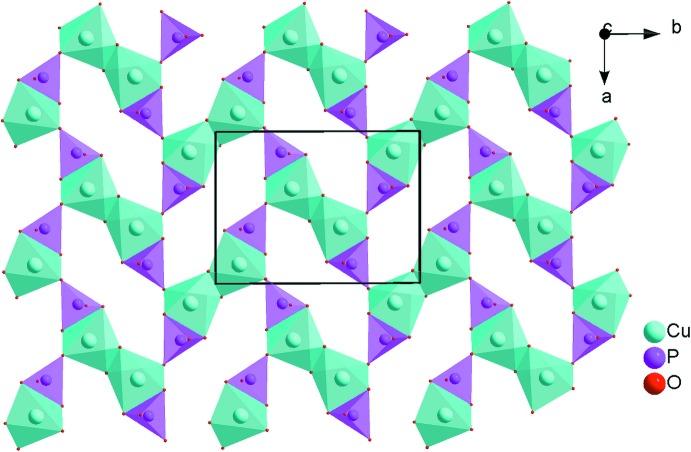
Projection along [001] of [Cu_2_P_2_O_12_] copper phosphate sheets in the crystal structure of NaCuIn(PO_4_)_2_.

**Figure 3:**
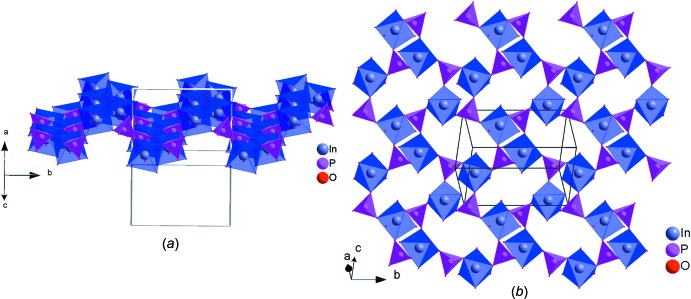
(*a*) A view approximately along [101] showing the undulating layer formed by [InO_6_] octa­hedra linked to PO_4_ tetra­hedra and (*b*) a projection of this layer onto (101).

**Figure 4:**
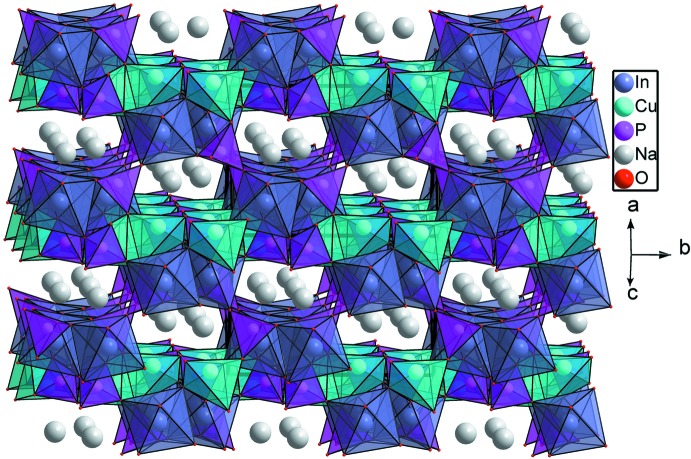
The sodium cations located in channels running parallel to [101] in the crystal structure of NaCuIn(PO_4_)_2_.

In this phosphate, two [CuO_5_] triangular bipyramids share one edge to form a [Cu_2_O_8_] dimer, the ends of which are linked to two P1O_4_ tetra­hedra by edge-sharing. The obtained [Cu_2_P_2_O_12_] groups are linked together *via* the vertices to form sheets extending parallel to (10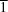), as shown in Fig. 2 ▸. On the other hand, the [InO_6_] octa­hedra and the P2O_4_ tetra­hedra are inter­connected through common vertices to build up an undulating layer extending in the same direction (Fig. 3 ▸). The copper phosphate layers are sandwiched between the undulating indium phosphate layers. By sharing corners and edges, an alternating stacking of the layers along [101] leads to a three-dimensional framework structure with channels in which the Na^+^ cations are located (Fig. 4 ▸). The four nearest oxygen atoms around the alkali metal cation form a distorted disphenoid with Na—O distances between 2.3213 (12) and 2.4275 (11) Å (Fig. 1 ▸).

NaCuIn(PO_4_)_2_ is isotypic with KCuFe(PO_4_)_2_ (Badri *et al.*, 2011 ▸), whereby potassium is substituted by sodium and iron by indium. However, we note a significant difference in the coordination number of sodium and potassium in the two structures. Whereas sodium has a fourfold coordination in NaCuIn(PO_4_)_2_, potassium is surrounded by nine oxygen atoms in KCuFe(PO_4_)_2_ because of its greater ionic radius.

Bond-valence-sum calculations (Brown & Altermatt, 1985 ▸) are in good agreement with the expected values (in valence units) for sodium(I), copper(II), indium(III) and the phospho­rus(V) cations, *viz*. Na^I^ = 0.845 (2), Cu^II^ = 2.102 (3), In^III^ = 3.152 (4), P1^V^ = 4.930 (8), and P2^V^ = 4.992 (8). For the oxygen anions, the calculated values range between 1.940 (5) and 2.076 (3).

## Database survey   

Phosphate-based materials with general formula *AM* ^II^ *M*′^III^(PO_4_)_2_ commonly show crystal structures where channels or, more rarely, layers are formed by the [*M* ^II^ *M*′^II^(PO_4_)_2_]^−^ framework to delimit suitable environments to accommodate the *A* ^+^ cations. A recent survey given by Yakubovich *et al.* (2019 ▸) revealed that all compounds of the morphotropic series *AM* ^II^ *M*′^III^(PO_4_)_2_, where *A* = Na, K, Rb or NH_4_, *M*′′ = Cu, Ni, Co, Fe, Zn or Mg and *M*′′′ = Fe, Al or Ga, crystallize in the monoclinic crystal system and can be classified into seven subgroups according to their structure types, *viz*. (i) KNiFe(PO_4_)_2_ (space-group type *P*2_1_/*c*, *Z* = 4; Strutynska *et al.*, 2014 ▸); (ii) KFe^II^Fe^III^(PO_4_)_2_ (space-group type *P*2_1_/*c*, *Z* = 4; Yakubovich *et al.*, 1986 ▸); (iii) (NH_4_)Fe^II^Fe^III^(PO_4_)_2_ (space-group type *C*2/*c*, *Z* =16; Boudin & Lii, 1998 ▸); (iv) K(Co,Al)_2_(PO_4_)_2_ (space-group type *C*2/*c*, *Z* = 8; Chen *et al.*, 1997 ▸); (v) (NH_4_)(Zn,Ga)_2_(PO_4_)_2_ (space-group type *P*2_1_/*a*, *Z* = 4; Logar *et al.*, 2001 ▸); (vi) KMgFe(PO_4_)_2_ (space-group type *C*2/*c*, *Z* = 4; Badri *et al.*, 2009 ▸); (vii) NaZnAl(PO_4_)_2_ (space-group type *P*2_1_/*c*, *Z* = 4; Yakubovich *et al.*, 2019 ▸). NaCuIn(PO_4_)_2_ belongs to the second subgroup of this classification.

In addition, the structures of certain members of this phosphate family are similar to those of the zeolite-*AB*W structural type (Badri *et al.*, 2014 ▸). When the trivalent cation is lanthanum or yttrium, the crystal structures K*M* ^II^La(PO_4_)_2_ (*M* ^II^ = Mg or Zn) are isotypes of the monazite monoclinic structure of LaPO_4_ with space-group type *P*2_1_/*n* (Pan *et al.*, 2006 ▸; Tie *et al.*, 1995 ▸), while KMgY(PO_4_)_2_ turns out to be an isotype of the xenotime structure YPO_4_ adopting a tetra­gonal symmetry with space-group type *I*4_1_/*amd* (Tie *et al.*, 1996 ▸).

## Synthesis and crystallization   

Stoichiometric amounts of NaNO_3_, CuO, In_2_O_3_ and NH_4_H_2_PO_4_ as precursors in the molar ratio 1:1:0.5:2 were ground in an agate mortar and pre-heated at 473 and 673 K in a platinum crucible to eliminate gaseous products. The resulting powder was subsequently heated to a temperature of 1473 K. The product was then cooled to room temperature at a rate of 5 K h^−1^. The obtained product contained green single crystals corresponding to the title phosphate.

## Refinement   

Crystal data, data collection and structure refinement details are summarized in Table 1 ▸.

Labelling of atoms and their coordinates were adapted from isotypic KCuFe(PO_4_)_2_ (Badri *et al.*, 2011 ▸). Since not all atoms in the latter description are part of one unit cell, a translation by (*z* + 1) relative to the original coordinates brings all corresponding atoms inside one unit cell. Moreover, oxygen atoms O11 and O14 were translated by (*x* − 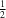, −*y* + 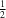, *z* − 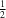) and (*x*, *y*, *z* − 1), respectively, to be linked directly to P1.

The maximum and minimum electron densities in the final difference-Fourier map are at 0.70 Å from O14 and 0.50 Å from Cu1, respectively.

## Supplementary Material

Crystal structure: contains datablock(s) I. DOI: 10.1107/S2056989020001929/wm5539sup1.cif

Structure factors: contains datablock(s) I. DOI: 10.1107/S2056989020001929/wm5539Isup2.hkl

CCDC reference: 1983244

Additional supporting information:  crystallographic information; 3D view; checkCIF report

## Figures and Tables

**Table 1:** Experimental details

Crystal data	
Chemical formula	NaCuIn(PO_4_)_2_
*M*_r_	391.29
Crystal system, space group	Monoclinic, *P*2_1_/*n*
Temperature (K)	296
*a*, *b*, *c* (Å)	8.2563 (3), 10.1382 (4), 8.8060 (3)
β (°)	114.444 (1)
*V* (Å^3^)	671.03 (4)
*Z*	4
Radiation type	Mo *K*α
μ (mm^−1^)	7.16
Crystal size (mm)	0.34 × 0.25 × 0.19

Data collection	
Diffractometer	Bruker X8 *APEX* Diffractometer
Absorption correction	Multi-scan (*SADABS*; Krause *et al.*, 2015 ▸)
*T*_min_, *T*_max_	0.528, 0.747
No. of measured, independent and observed [*I* > 2σ(*I*)] reflections	24292, 3106, 2996
*R*_int_	0.026
(sin θ/λ)_max_ (Å^−1^)	0.820

Refinement	
*R*[*F* ^2^ > 2σ(*F* ^2^)], *wR*(*F* ^2^), *S*	0.013, 0.033, 1.11
No. of reflections	3106
No. of parameters	119
Δρ_max_, Δρ_min_ (e Å^−3^)	0.66, −0.59

Computer programs: *APEX2* and *SAINT* (Bruker, 2009 ▸), *SHELXT2014/5* (Sheldrick, 2015*a* ▸), *SHELXL2018/3* (Sheldrick, 2015*b* ▸), *ORTEP-3 for Windows* (Farrugia, 2012 ▸), *DIAMOND* (Brandenburg, 2006 ▸) and *publCIF* (Westrip, 2010 ▸).

## supplementary crystallographic information

### Crystal data

**Table:**

NaCuIn(PO_4_)_2_	*F*(000) = 732
*M**_r_* = 391.29	*D*_x_ = 3.873 Mg m^−^^3^
Monoclinic, *P*2_1_/*n*	Mo *K*α radiation, λ = 0.71073 Å
*a* = 8.2563 (3) Å	Cell parameters from 3106 reflections
*b* = 10.1382 (4) Å	θ = 2.9–35.6°
*c* = 8.8060 (3) Å	µ = 7.16 mm^−^^1^
β = 114.444 (1)°	*T* = 296 K
*V* = 671.03 (4) Å^3^	Block, green
*Z* = 4	0.34 × 0.25 × 0.19 mm

### Data collection

**Table:**

Bruker X8 APEX Diffractometer	3106 independent reflections
Radiation source: fine-focus sealed tube	2996 reflections with *I* > 2σ(*I*)
Graphite monochromator	*R*_int_ = 0.026
φ and ω scans	θ_max_ = 35.6°, θ_min_ = 2.9°
Absorption correction: multi-scan (SADABS; Krause *et al.*, 2015)	*h* = −12→13
*T*_min_ = 0.528, *T*_max_ = 0.747	*k* = −16→16
24292 measured reflections	*l* = −14→14

### Refinement

**Table:**

Refinement on *F*^2^	0 restraints
Least-squares matrix: full	*w* = 1/[σ^2^(*F*_o_^2^) + (0.0139*P*)^2^ + 0.5758*P*] where *P* = (*F*_o_^2^ + 2*F*_c_^2^)/3
*R*[*F*^2^ > 2σ(*F*^2^)] = 0.013	(Δ/σ)_max_ = 0.004
*wR*(*F*^2^) = 0.033	Δρ_max_ = 0.66 e Å^−^^3^
*S* = 1.11	Δρ_min_ = −0.59 e Å^−^^3^
3106 reflections	Extinction correction: SHELXL-2018/3 (Sheldrick, 2015b), Fc^*^=kFc[1+0.001xFc^2^λ^3^/sin(2θ)]^-1/4^
119 parameters	Extinction coefficient: 0.0093 (3)

### Special details

**Table:**

Geometry. All esds (except the esd in the dihedral angle between two l.s. planes) are estimated using the full covariance matrix. The cell esds are taken into account individually in the estimation of esds in distances, angles and torsion angles; correlations between esds in cell parameters are only used when they are defined by crystal symmetry. An approximate (isotropic) treatment of cell esds is used for estimating esds involving l.s. planes.

### Fractional atomic coordinates and isotropic or equivalent isotropic displacement parameters (Å^2^)

**Table:**

	*x*	*y*	*z*	*U*_iso_*/*U*_eq_	
Na1	0.51418 (10)	−0.16856 (7)	1.09748 (8)	0.01965 (13)	
Cu1	0.37225 (2)	0.11940 (2)	0.45881 (2)	0.00706 (3)	
In1	0.00214 (2)	0.12812 (2)	0.73403 (2)	0.00463 (3)	
P1	0.12997 (4)	0.17027 (3)	0.15664 (4)	0.00494 (5)	
O11	−0.03216 (13)	0.25215 (10)	0.13588 (12)	0.01037 (15)	
O12	0.30223 (12)	0.24790 (9)	0.27141 (11)	0.00812 (14)	
O13	0.14612 (12)	0.04925 (9)	0.27222 (11)	0.00824 (14)	
O14	0.13759 (14)	0.12965 (10)	−0.00448 (12)	0.01159 (16)	
P2	0.28460 (4)	−0.08358 (3)	0.66933 (4)	0.00448 (5)	
O21	0.11495 (13)	−0.13653 (9)	0.52826 (12)	0.00993 (15)	
O22	0.37706 (12)	−0.19241 (8)	0.79630 (11)	0.00787 (14)	
O23	0.24068 (13)	0.03111 (9)	0.75876 (12)	0.00903 (15)	
O24	0.41607 (13)	−0.03247 (9)	0.59836 (12)	0.00962 (15)	

### Atomic displacement parameters (Å^2^)

**Table:**

	*U*^11^	*U*^22^	*U*^33^	*U*^12^	*U*^13^	*U*^23^
Na1	0.0288 (3)	0.0145 (3)	0.0146 (3)	−0.0042 (2)	0.0079 (3)	−0.0035 (2)
Cu1	0.00887 (7)	0.00557 (6)	0.00492 (6)	−0.00104 (4)	0.00104 (5)	0.00126 (4)
In1	0.00517 (4)	0.00405 (4)	0.00432 (4)	−0.00022 (2)	0.00161 (3)	−0.00042 (2)
P1	0.00552 (11)	0.00490 (11)	0.00367 (11)	−0.00020 (9)	0.00119 (9)	0.00053 (8)
O11	0.0096 (4)	0.0125 (4)	0.0094 (4)	0.0054 (3)	0.0044 (3)	0.0042 (3)
O12	0.0085 (4)	0.0077 (3)	0.0063 (3)	−0.0034 (3)	0.0012 (3)	0.0008 (3)
O13	0.0091 (4)	0.0055 (3)	0.0071 (3)	−0.0028 (3)	0.0004 (3)	0.0020 (3)
O14	0.0129 (4)	0.0165 (4)	0.0043 (3)	0.0025 (3)	0.0025 (3)	−0.0014 (3)
P2	0.00536 (11)	0.00392 (11)	0.00410 (11)	0.00089 (8)	0.00189 (9)	0.00025 (8)
O21	0.0096 (4)	0.0119 (4)	0.0054 (3)	−0.0026 (3)	0.0002 (3)	−0.0013 (3)
O22	0.0109 (4)	0.0058 (3)	0.0073 (3)	0.0032 (3)	0.0041 (3)	0.0027 (3)
O23	0.0088 (4)	0.0071 (3)	0.0113 (4)	0.0018 (3)	0.0042 (3)	−0.0034 (3)
O24	0.0101 (4)	0.0092 (4)	0.0125 (4)	0.0030 (3)	0.0077 (3)	0.0056 (3)

### Geometric parameters (Å, º)

**Table:**

Na1—O21^i^	2.3213 (12)	In1—O22^viii^	2.1441 (9)
Na1—O23^ii^	2.3496 (12)	In1—O13^vii^	2.1632 (9)
Na1—O22	2.4268 (11)	In1—O12^ix^	2.2051 (9)
Na1—O11^iii^	2.4275 (11)	P1—O14	1.5035 (10)
Cu1—O24	1.9088 (9)	P1—O11	1.5205 (10)
Cu1—O11^iv^	1.9317 (9)	P1—O13	1.5642 (9)
Cu1—O12	1.9913 (9)	P1—O12	1.5729 (9)
Cu1—O13	2.0378 (9)	P2—O23	1.5297 (9)
Cu1—O24^v^	2.1939 (9)	P2—O22	1.5310 (9)
In1—O14^vi^	2.1028 (10)	P2—O21	1.5340 (10)
In1—O21^vii^	2.1044 (9)	P2—O24	1.5488 (9)
In1—O23	2.1303 (9)		
			
O21^i^—Na1—O23^ii^	108.90 (4)	O14^vi^—In1—O13^vii^	94.18 (4)
O21^i^—Na1—O22	71.58 (4)	O21^vii^—In1—O13^vii^	90.44 (4)
O23^ii^—Na1—O22	123.80 (4)	O23—In1—O13^vii^	96.21 (4)
O21^i^—Na1—O11^iii^	94.99 (4)	O22^viii^—In1—O13^vii^	171.80 (3)
O23^ii^—Na1—O11^iii^	88.91 (4)	O14^vi^—In1—O12^ix^	85.65 (4)
O22—Na1—O11^iii^	146.95 (4)	O21^vii^—In1—O12^ix^	96.23 (4)
O24—Cu1—O11^iv^	96.82 (4)	O23—In1—O12^ix^	164.87 (3)
O24—Cu1—O12	166.88 (4)	O22^viii^—In1—O12^ix^	87.16 (3)
O11^iv^—Cu1—O12	96.28 (4)	O13^vii^—In1—O12^ix^	91.55 (3)
O24—Cu1—O13	95.96 (4)	O14—P1—O11	114.50 (6)
O11^iv^—Cu1—O13	144.90 (4)	O14—P1—O13	111.94 (5)
O12—Cu1—O13	72.85 (3)	O11—P1—O13	109.89 (5)
O24—Cu1—O24^v^	82.35 (4)	O14—P1—O12	111.32 (5)
O11^iv^—Cu1—O24^v^	110.97 (4)	O11—P1—O12	108.72 (5)
O12—Cu1—O24^v^	93.34 (4)	O13—P1—O12	99.40 (5)
O13—Cu1—O24^v^	103.05 (4)	O23—P2—O22	108.94 (5)
O14^vi^—In1—O21^vii^	174.97 (4)	O23—P2—O21	110.57 (5)
O14^vi^—In1—O23	80.87 (4)	O22—P2—O21	110.58 (5)
O21^vii^—In1—O23	96.68 (4)	O23—P2—O24	107.97 (5)
O14^vi^—In1—O22^viii^	93.79 (4)	O22—P2—O24	108.36 (5)
O21^vii^—In1—O22^viii^	81.66 (3)	O21—P2—O24	110.35 (5)
O23—In1—O22^viii^	86.94 (4)		

Symmetry codes: (i) *x*+1/2, −*y*−1/2, *z*+1/2; (ii) −*x*+1, −*y*, −*z*+2; (iii) −*x*+1/2, *y*−1/2, −*z*+3/2; (iv) *x*+1/2, −*y*+1/2, *z*+1/2; (v) −*x*+1, −*y*, −*z*+1; (vi) *x*, *y*, *z*+1; (vii) −*x*, −*y*, −*z*+1; (viii) −*x*+1/2, *y*+1/2, −*z*+3/2; (ix) *x*−1/2, −*y*+1/2, *z*+1/2.
